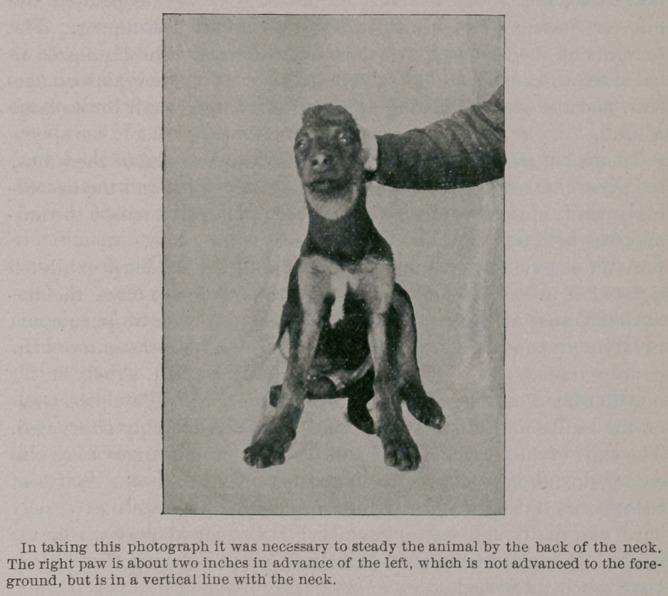# A Case of Acromegaly in a Dog

**Published:** 1897-07

**Authors:** R. H. Cunningham

**Affiliations:** Assistant Demonstrator in Physiology, College of Physicians and Surgeons, Columbia University, New York


### A CASE OF ACROMEGALY IN A DOG.

By R. H. Cunningham, M.D.,
ASSISTANT DEMONSTRATOR IN PHYSIOLOGY, COLLEGE OF PHYSICIANS AND SURGEONS, COLUMBIA UNIVERSITY, NEW YORK.

Since the publication in 1886 of Marie's[1] description of two cases of the disease to which he applied the name "acromégalie," over a hundred more or less authenticated cases have been described as occurring in man. That this disease might affect in a more or less

[1] P. Marie: Revue de Medicine, vi, Paris, 1886, p. 297.

typical manner some of the orders of the lower animals has no doubt occurred to many observers ere this; but I have not been able to find in any of the medical or veterinary literature any report of this disease having been observed in the lower animals. Consequently, the case of a dog described in this article is all the more interesting, and, as far as I am aware, extremely unique.

Briefly, this dog, whose photograph is reproduced in the accompanying illustration, came under my observation in the Physiological Department of Columbia University at the College of Physicians and Surgeons, New York, during the latter part of

In taking this photograph it was necessary to steady the animal by the back of the neck. The right paw is about two inches in advance of the left, which is not advanced to the foreground, but is in a vertical line with the neck.

May, 1895, and was such an extraordinary looking beast that I determined to keep the animal for a number of weeks and observe him. Apparently, this animal was a well-muscled member of a mongrel breed, probably a mixture of the black-and-tan and the hound. Its permanent canine teeth were well developed, and its age was very probably over a year. Its weight was 13.25 kilos. A more sluggish and lazier canine specimen I have never seen. To anyone familiar with the clinical picture of acromegaly in man, the most superficial examination of the animal would instantly remind one of that disease, especially when due allowance was made for the differences between the human facies and the canine facies. The markedly enlarged forefeet, with thickened phalanges, bearing claws apparently too small for them, and the broad face, with the lower incisors overlapping the upper, were very striking and certainly very forcibly recalled the clinical picture of acromegaly. The hind feet were not quite so enlarged in proportion to the size of the leg, but both the fore and the hind feet were considerably broadened and thickened, both the soft parts and the bones appearing to participate in the enlargement. Prognathous was not noticeable, but when the lips of the animal were separated the inferior incisor teeth considerably overlapped the upper. The margins of the orbits, the malar, and the nasal bones appeared to be much thickened. The palpebral fissure of both eyes was narrow, and the eyes of the dog appeared extremely small for so large a head.

In spite of the apparent development of the muscles of the limbs, the general strength of the animal was diminished, and the exceedingly weak muscular efforts became very perceptible when the animal was held and kept from moving about the room.

After observing the animal for three weeks it suddenly exhibited signs of a bronchitis and died in the course of two days, the immediate cause of death proving to be an acute broncho-pneumonia involving a considerable portion of both lungs. At the autopsy the costal cartilages were found to be partially ossified, chiefly in the neighborhood of the costo-chondral articulations. The lower ends of the radius and ulnar were enlarged and considerably eburnated. The ends of metacarpal bones and the ends of phalanges were also enlarged and very markedly eburnated. Although the shafts of these bones did not seem to be much enlarged, they were extremely hard externally and quite vascular in the central portions of the bone. The periosteum and other soft parts covering these bones were much thickened.

The thyroid gland was the seat of an interstitial thyroiditis, and both lobes were markedly reduced in size. The right lobe weighed but 0.5 gramme and the left 1.1 gramme. No accessory thyroid bodies could be detected in any of the situations in which they frequently occur. The right thyroid lobe, as one would expect from the above-mentioned weight, was more indurated than the left, but even the latter was so indurated that when it was being cut one could easily imagine that a firm fibrous tendon was being divided by the knife.

The thymus gland was not only present, but was of considerable size, weighing 10.80 grammes after the surrounding fat had been removed as closely as possible. Sections of the gland under the microscope showed that in nearly all parts the structure was identical with that of developing thymus, in which the ramifying acini contain numbers of nucleated epithelial cells. Great vascularity of the gland was also very noticeable.

On attempting to trephine the skull preparatory to removing the calvarium with the rongeur forceps it was found that the bones of the skull were much thickened and possessed extremely hard and brittle internal and external tables. For instance, at the left parietal protuberance the parietal bone was 6 mm. in thickness. The diploë of the thickened bones was very vascular and was readily cut by the forceps after the hard outer table had been broken. The brain and spinal cord exhibited no perceptible changes, but the condition of the hypophysis was interesting. It was very vascular and œdematous, being very similar in appearance to that of a dog that has survived the removal of the thyroid body for five or six days. As the writer had not at that time compared the weight and the dimensions of the dog's pituitary body with the body-weight, it was difficult to affirm whether or not the pituitary in this case was in reality enlarged. Since that animal's death, however, a number of such comparisons have been made on dogs of about the same weight as that dog, and from the results of these comparisons I judge that the pituitary body of this dog was about one-third larger than that of a normal dog of about the same weight. Under the microscope the structure of the pituitary body and its mantel did not very profoundly differ from that of the pituitary of a normal dog. In the former preparations, however, the arrangement of the epithelium into fairly well differentiated acini, which frequently possessed a small lumen surrounded by cylindrical or by polygonal cells, was more decided than in the hypophysis of the normal dog. The small cysts were more numerous also, and some of them contained a small amount of colloid. An increased vascularity of the body was also noticeable.

The heart, spleen, and suprarenal bodies appeared to be normal. Broncho-pneumonia was thus the immediate cause of death.

When the above-mentioned pathological changes in the thyroid gland, the hypertrophy of the thymus gland, the eburnation and the enlargement of the bones, and, lastly, the moderate enlargement of the hypophysis are considered, I think that there is scarcely any doubt that this dog was a true example of canine acromegaly.

Until the autopsy it was impossible to make more than a provisional diagnosis, for it is well known that some growing young dogs frequently have large feet, long legs, broad heads, and bodies that appear quite out of proportion to their legs. Such dogs, however, do not present marked interstitial changes in the thyroid gland, hypertrophied thymus glands, and thickened and eburnated facial and cranial bones. Consequently they may be excluded. Similarly, canine rickets, the only other disease that I know which can produce in the dog a clinical picture much resembling that exhibited by the dog described in this paper, can be excluded, owing to the character of the changes found in the bones. Before the autopsy it must be admitted that it was impossible to exclude rickets; but as soon as the thickened, eburnated bones were cut, sawn, and otherwise examined it was evident that the osseous changes were of a totally different kind to those so characteristic of rhachitic bones. Changes in the thyroid gland have been noted by Trachewsky, Lanz, and others, in rickets, but such a profound degree of thyroid atrophy, accompanied by hypertrophy of the hypophysis and by hypertrophy of the thymus gland, was not detected by any of those writers.

It is thus evident that acromegaly was about the only disease that could be accompanied by the above briefly mentioned symptoms and pathological alterations, and undoubtedly the animal was a genuine example of canine acromegaly.

---

A decoction of tobacco is in use in one of the experiments now under trial for dipping sheep.

Officials of the Belgian army are making inquiries among our horse-breeding centres for horses suitable for military service.

Bits to prevent cows from sucking their own milk are now in successful use.

Great Britain now demands that all dogs intended for shipment to that country must first obtain a license from the Board of Agriculture at 4 Whitehall Place, London, S. W.

Japan has become a buyer of our horses, large consignments being made from Seattle, Washington.

Harrison county, Kentucky, is the seat of an outbreak of glanders now being inspected by the State veterinarian. Eight horses have already been destroyed. The State allows compensation for all animals destroyed.

## Figures and Tables

**Figure f1:**